# The Role of Narrow Band Imaging in the Detection of Recurrent Laryngeal and Hypopharyngeal Cancer after Curative Radiotherapy

**DOI:** 10.1155/2014/175398

**Published:** 2014-06-30

**Authors:** Michal Zabrodsky, Petr Lukes, Eva Lukesova, Jan Boucek, Jan Plzak

**Affiliations:** Department of Otorhinolaryngology and Head and Neck Surgery, 1st Faculty of Medicine, Charles University, University Hospital Motol, V Úvalu 84, 150 06 Prague 5, Czech Republic

## Abstract

Narrow band imaging is considered a significant improvement in the possibility of detecting early mucosal lesion of the upper aerodigestive tract. Early detection of mucosal neoplastic lesions is of utmost importance for patients survival. There is evidence that, especially in patients previously treated by means of curative radiotherapy or chemoradiotherapy, the early detection rate of recurrent disease is quite low. The aim of this study was to prove whether the videoendoscopy coupled with NBI might help detect recurrent or secondary tumors of the upper aerodigestive tract. 66 patients previously treated by means of RT or CRT with curative intent were enrolled in the study. All patients underwent transnasal flexible videoendoscopy with NBI mode under local anesthesia. When a suspicious lesion was identified in an ambulatory setting, its nature was proved histologically. Many of these changes were not identifiable by means of conventional white light (WL) endoscopy. The accuracy, sensitivity, specificity, and positive and negative predictive value of the method are very high (88%, 92%, 76%, 96%, and 91%, resp.). Results demonstrate that outpatient transnasal endoscopy with NBI is an excellent method for the follow-up of patients with carcinomas of the larynx and the hypopharynx primarily treated with radiotherapy.

## 1. Introduction

Malignant tumors of the larynx account for approximately 30% of all malignant tumors in the head and neck region. The most common histological type is squamous cell carcinoma. Early diagnosis of a primary tumor in this location affects the possibility of maintaining the main functions of the larynx, in particular respiration, protection of the airway against the aspiration of food, and also phonation. Only tumors at an early stage can be treated with organ preserving surgical techniques or single nonsurgical oncological treatment modality. More advanced tumors are usually treated by a combination of methods. A therapeutic failure will result in the local relapse of disease. The time factor plays a crucial role in the possibility of application of the organ-saving protocols. The early detection of relapse dramatically affects the prognosis of patients [1]. Follow-up of patients with tumors of the larynx and hypopharynx can be very complicated due to the complex anatomy of this region. Transnasal flexible videoendoscopy is the best method for observation of these patients. New chip-on-the-tip videoendoscopes with the option of obtaining images in HD quality provide the best vision of the observed region. The identification of suspect lesions can be obscured by the effects of radiotherapy (RT) or chemoradiotherapy (CRT). This aggressive treatment leads to changes of mucosa, such as signs of chronic inflammation with hypervascularization and swelling. Another negative byproduct of radiotherapy is reduced saliva production by minor salivary glands, which leads to the secretion of viscous mucous [2]. The mucose membrane in the irradiated area may also be more sensitive to further damage including the effects of smoking or laryngopharyngeal reflux. All these changes can mask the recurrence of a primary tumor or the identification of a secondary tumor in the same location. Therefore, in our institution microlaryngoscopy under general anesthesia was part of the follow-up protocol. The examination under general anesthesia allows very careful inspection of mucosal surfaces including the possibility to palpate suspect areas and perform a very precisely targeted biopsy.

The narrow-band imaging (NBI) is a relatively new optical method which has some very unique features. The method is based on an illumination of the mucosal surface by light with a defined narrow band of wavelengths. Two narrow bands centered on 415 (blue light) and 540 nm (green light) are used in the system provided by Olympus (Olympus Medical Corporation, Tokyo, Japan). These selected wavelengths can pass through the mucosa to a defined depth and also correlate with absorption maxims of a molecule of hemoglobin. A wavelength of 415 nm penetrates only the very superficial layers of the mucosa and is absorbed by blood containing intrapapillary capillary loops (IPCL). The narrow band centered on 540 nm penetrates the deeper level and accentuates the venules and arterioles located under a layer of IPCL. The illumination of the mucosal membrane in the NBI mode therefore tends to substantially increase the contrast between blood containing vessels and surrounding tissues. Therefore, even very subtle changes in microvascular architecture can be identified. Microvascular changes are typical for dysplastic squamous epithelium lesions and early stage squamous cell carcinomas as described in numerous studies [3]. Prolongation of the capillary loops occurs at the beginning, gradually the caliber of IPCL enlarges, and the capillary loops begin to have a winding shape as the dysplastic lesion continues to progress. The ultimate stage of these processes is the utter destruction of the physiological microvascular arrangement. Due to the abovementioned hypervascularization of the irradiated mucose membrane, the detection of changes in capillary architecture is much more difficult even with the NBI mode employment (Figure 2). The epithelium at the site of the primary tumor is destroyed by external beam radiation to such an extent that a normal arrangement of capillary loops may no longer be evident. The presence of pathological IPCL can then be very sporadic. In this situation clinicians need to also take into account other signs indicating the presence of a tumor. These may be, for example, a pronounced and irregular feeding vessel, the presence of superficial necrosis, ulceration, and so forth. Therefore, the aim of this study was to determine the effectiveness of the NBI in follow-up of patients previously treated with radiotherapy or chemoradiotherapy with a curative intent. Defining the accuracy of the detection of recurrences or onset of secondary tumors of the mucosal surface was the main goal of the research.

## 2. Material and Methods

The presented study was conceived as a prospective study. 66 consecutive patients previously treated by means of RT or CRT with a curative intent for a primary cancer of the larynx and hypopharynx and followed up in the period from January 2010 to January 2014 at a single tertiary institution were enrolled in the study. Patients were included in the study after signing informed consent. All patients were regularly followed up and had a minimum of two videoendoscopic examinations; the first was in the timeframe of 4–12 weeks upon completion of radiotherapy, and the next was scheduled three months afterward. With a longer interval from the end of the oncological therapy, the time between the follow-up visits gradually increased. The exceptions were patients with suspicious findings at first follow-up visit. These were scheduled directly for examination under general anesthesia. All examinations were systematically carried out by the same schema as described below (see Table 1).

All patients underwent transnasal flexible videoendoscopy under local anesthesia. NBI mode was used in every evaluation. Changes in microvascular architecture of the mucosa were classified according to Ni's classification [4] of microvascular endoscopic patterns in superficial mucosal lesions. We always identified microvascular changes typical of tumor growth in patients rated as TP, but if their presence was scant, we focused also on the other features of tumor growth mentioned in Section 1. Whenever a suspicious lesion was identified in an ambulatory setting, its nature was proven histologically. Findings were classified as true positive (TP) if the histological analysis demonstrated anything from severe mucosal dysplasia to infiltrative squamous cell cancer. Patients with persistently negative findings were considered true negatives (TN). The rigid endoscopy with white light WL and NBI HDTV was performed to prove the nature of every suspect lesion. The cases were considered false positive (FP) whenever histology did not reveal the cancer at the area marked by the previous videoendoscopy examination. Finally, cases with pathologic lesions identified in the WL and NBI magnifying endoscopy but not in the outpatient WL and NBI flexible videoendoscopy were considered false negatives (FN).

The study was approved by the Ethics Committee of the Faculty Hospital Motol and written informed consent was obtained from all the patients participating in the study before the endoscopic examination and further treatment commenced.

### 2.1. Videoendoscopy Equipment and Procedure

All patients were examined using the Evis Exera II optical system with CLV-180B light source (Olympus Medical Systems, Tokyo, Japan). A flexible videolaryngoscope Olympus ENF VQ (Olympus Medical Systems, Tokyo, Japan) was used in outpatient settings. All examinations were recorded unedited on the recording device in AVI format and stored for subsequent analysis. The vast majority of patients were inspected under topical anesthesia. Patients were examined in the upright position. Local anesthesia was induced by spraying Xylocaine 10% (lidocaine hydrochloride) intranasally and then transorally on the mucosa of the nasal cavity, oro- and hypopharynx, and the larynx. All testing was performed by three experienced otolaryngologists (Michal Zabrodsky, Petr Lukes, and Eva Lukesova). The examination in WL was carried out first, followed by the endoscopy in the NBI mode. A suspect lesion was defined in line with previous practice and knowledge as a well-demarcated brownish mucosal lesion with clearly visible pathologic IPCL. The recordings were subsequently evaluated by a pair of laryngologists (Michal Zabrodsky and Petr Lukes); the findings were evaluated as negative or positive only in case both concurred on the final evaluation. In case of disagreement in the assessment, the opinion of the physician performing the endoscopy was taken into account. In the case of a positive evaluation of the lesion, the patient was subsequently sent for the diagnostic or therapeutic assessment in direct laryngoscopy with microscopic assessment of laryngeal mucosa and WL and NBI HDTV telescopic examination. For the direct endoscopy angled telescopes (0, 30, and 70° angles of view) (Olympus Medical Systems, Tokyo, Japan) were employed. The examination was again performed in the white light and the NBI mode. The findings were recorded. All suspect mucosal lesions were eventually resected completely through endoscopically assisted surgery or a biopsy of a substantial portion of the lesion was performed to obtain a representative specimen in case it was not possible to make the diagnosis and treatment during the same session. The biopsy was also made from any other suspect lesion indicated by WL and NBI intraoperative HDTV magnifying endoscopy. The results of optical methods were then compared with the results of histological analysis. On the basis of a comparison of the results, the data was statistically processed and sensitivity, specificity, and positive and negative predictive value of outpatient videoendoscopic evaluation were determined. Suspected findings in other head and neck regions than the larynx and hypopharynx were not included in the final assessment of the effectiveness of the method.

## 3. Results

The average age of the patients was 63 years (range: 42–83 years); there was a prevalence of men (ratio of 44 men : 12 women). The vast majority of primary tumors were at the glottic region (*N* = 56. 85%), supraglottic (*N* = 5. 8%), hypopharyngeal (*N* = 4. 6%), and subglottic tumors accounted for a minority of patients. 38 patients were classified as an early-staged primary tumor (T1a or T1b), 19 patients had tumor stage T2, and the remaining 9 patients had tumors classified as stage T3. 13 patients (20%) had initial endoscopic treatment of the disease; nonsurgical oncological therapy had been indicated due to the impossibility of complete resection of the tumor or because patients rejected radicalization procedures. Five patients (8%) were treated by a combined treatment of external beam radiotherapy and chemotherapy, and one (2%) received a combination of external beam radiotherapy and biological therapy. All the other patients were treated with radiotherapy only with a maximum dose of radiation focused on the region of the larynx and hypopharynx. All patients were treated exclusively by intensity modulated radiotherapy. The average duration of follow-up was 31.3 months (range: 12–48, median: 27 months)—see also Table 2.

The transnasal videoendoscopic evaluation with NBI system revealed a suspected relapse of the disease or secondary tumor in 17 patients (Figures 3 and 4); 3 cases were already highly suspicious during an outpatient WL videoendoscopy. Suspected mucosal lesion, which was not detectable in the videoendoscopic testing in WL, was detected in 14 patients (21%). In all the 17 patients pathological lesions were also rated as suspicious during intraoperative WL and NBI magnifying endoscopy. Moreover, two additional pathologic lesions were identified only upon WL and NBI magnifying endoscopy. We were not able to sufficiently expose the larynx in one patient due to unfavorable anatomical conditions—trismus, scoliosis, and high denture—and so the value of endoscopy was limited. Therefore the biopsy was performed under topical anesthesia in the outpatient office. We were able to prove histologically carcinoma in situ in this patient. The patient subsequently underwent an external approach cordectomy. However, final histological analysis failed to show a relapse of the original disease. All other lesions have been addressed by attempting a radical endoscopic removal; in cases deemed not suitable for an endoscopic approach, a representative tissue sample was obtained from the tumorous tissue and this was sufficient to confirm the presence of a recurrent tumor. Histological analysis confirmed the malignant nature of the suspicious lesion in 15/19 patients (79%); in 4 cases despite the suggested findings of the endoscopic evaluation, the histology proved negative (21%). Characteristics of TP and FP cases are described in Tables 3 and 4. The majority of accurately diagnosed tumor recurrences were identified within three-year period from the termination of the primary treatment. Three of them were identified within the first six months—see Figure 5.

With the WL and NBI magnifying endoscopy we found more than one lesion in the region of laryngeal and hypopharyngeal mucosa in two patients and both of them were considered FN in relation to the result of outpatient videoendoscopy and TP in relation to the result of the magnifying endoscopy. So in total we identified 68 lesions. In 4 patients with clinical suspicion, histological analysis did not reveal any cancerous changes. These were consequently identified as false positive (FP; 4/19; 21%). The previously mentioned patient with histologically proven carcinoma in situ obtained from biopsy taken under local anesthesia, which subsequently underwent the cordectomy from the external approach with negative histology, was still considered a positive identification of the relapse of the disease and was rated as one of the TP. Two cases with more than one pathologic mucosal lesion (the second lesion unidentifiable by the outpatient videoendoscopy) were considered FN for outpatient videoendoscopy and TP for magnifying endoscopy. The remaining 49 patients with repeatedly negative results of videoendoscopic findings were considered to be actually negative (TN; 49/68; 72%). The sensitivity, specificity, and positive and negative predictive values of the ambulatory videolaryngoscopy were calculated from the obtained values and are shown in Table 5.

## 4. Discussion

Patients with any type of head and neck cancer after nonsurgical oncological treatment are sometimes difficult cases to follow up and it is frequently very difficult to detect an eventual relapse of the original disease or the presence of a secondary metachronous tumor [5–7]. As already mentioned, the oncological therapy has many undesirable side effects that change the quality of tissues and make the identification of tumorous changes challenging [2]. Late diagnosis of recurrence or secondary tumor results in deterioration in the default position not only for the preservation of function of the affected organ, but also for the final overall survival of patients [8]. In particular oncological therapy of extensive tumors leads to massive qualitative changes of tissues in the original site of the primary tumor. Basically it is difficult to decide what the real disease relapse is and what just abnormal trophicity of the soft tissues related to the therapy is. These changes can occur during therapy and persist for a relatively long period of time. In patients with recurrent disease it is still possible to perform a salvage surgery with the intention of preserving organ function, but in up to 50% of patients total laryngectomy cannot be avoided [8]. The time factor for the detection of persistent or recurrent disease plays a crucial role. The literature also mentioned the risk of necrosis of the thyroid cartilage associated with an early biopsy in suspected recurrence of the tumor [9]. It should be noted that cartilage necrosis is a rare complication of radiotherapy (appears with a frequency of around 1%) [7] and is more dependent on the total dose of radiation (at a total dose greater than 70 Gy) [10] or a recurrence of the original disease. The actual biopsy is not, in our opinion, the cause of necrosis, unless the biopsy creates deep defect extending into the cartilage.

The diagnostics of mucosal cancerous changes has undergone significant development in recent decades. Of noninvasive diagnostic methods ^18^FDG-PET scans are among the few options capable of identifying early recurrences or secondary tumor occurrence [11]. This examination is considered relatively less precise in the period of the first 6–8 weeks after the completion of cancer therapy, because the vascular supply of tissues is still altered and signs of RT-induced inflammation persist. However, the method is attributed to very high specificity, sensitivity, negative predictive value, and slightly lower positive predictive value. In a study carried out by Kostakoglu et al., these values (sensitivity, specificity, negative predictive value, and positive predictive value) reached 100%, 87%, 100%, and 56%, respectively [12]. In their study the first ^18^FDG-PET examination with high resolution computed tomography (HRCT) was performed at intervals of approximately 3.5 months after the completion of the therapy; the median of the radiological diagnosis of persistence or recurrence of the tumor was 6 months. From this perspective, ^18^FDG-PET would surely fulfill its purpose; however, the financial aspect comes into play. It goes without saying that this method is hardly possible to perform in short intervals due the financial expense. Authors advocating ^18^FDG-PET stress the difficulty of performing endoscopic examination, especially for the persistent presence of chronic inflammatory changes and postactinic edema. Conversely the method certainly does not have enough resolution to detect small superficial mucosal lesions, which are most suitable for endoscopically assisted surgery or partial procedures on the larynx. In another paper, Zbären et al. demonstrated that the accuracy of the correct determination of the extent of recurrent tumors of the larynx or hypopharynx in patients primarily treated with radiotherapy is very unsatisfactory [13]. They studied 42 patients with recurrent tumor in the region of the larynx, who underwent salvage surgical therapy. The precision of the determination of the extent of relapse using indirect endoscopes, microlaryngoscopy, and imaging methods was calculated. The precision was remarkably low at only 50% for endoscopic techniques and dropped to 24% for radiological studies. If the patient is planned for total laryngectomy, these diagnostic errors do not play as great a role as when planning partial or even endoscopic procedures. The importance of achieving an accurate and timely diagnosis is expressed by a modest success rate of endoscopic surgery or partial laryngectomy in patients with local recurrences.  Table 3 shows that the larynx preservation rate of the rescue of the larynx was 5/14 (37%). One of the patients treated endoscopically had subsequent relapse of the disease and died of disease persistence. Another patient scheduled for partial laryngectomy died of cardiopulmonary arrest. All remaining patients have no signs of recurrence of the original disease at the time of analysis.

**Figure 1:**
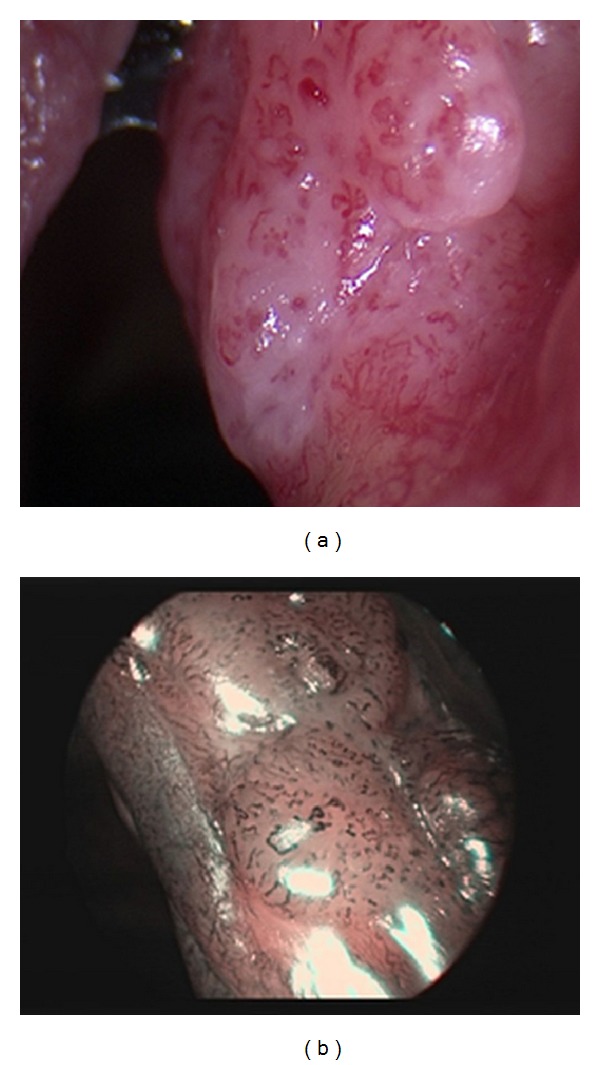
(a) SCC in WL examination. (b) NBI examination—well-demarcated brownish area with thick brown dots.

**Figure 2:**
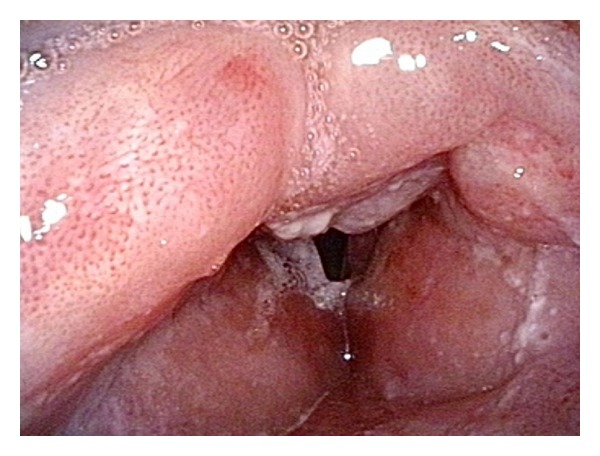
Late RT-related changes of the laryngopharyngeal mucosa. Note diffuse and regular distribution of IPCL.

**Figure 3:**
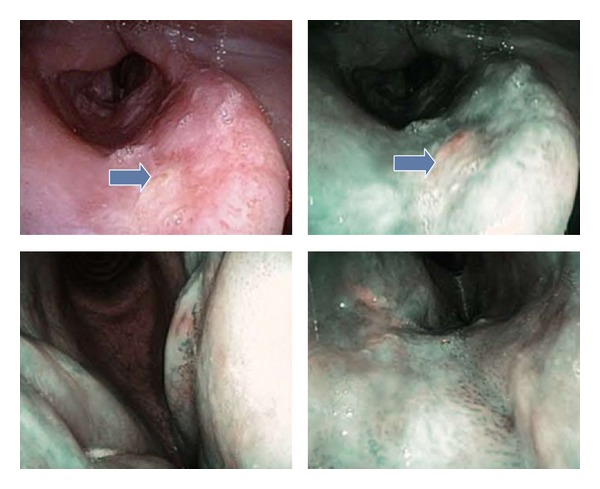
Appearance of the histologically proven invasive SCC, recurrence after RT.

**Figure 4:**
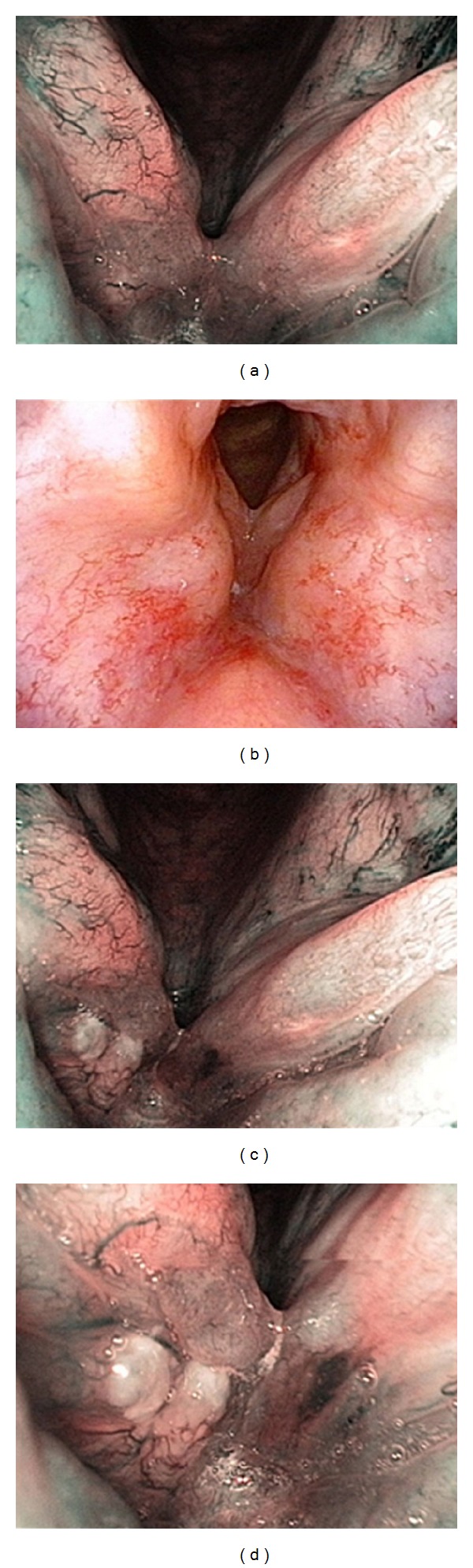
(a and b) Videoendoscopic aspect at the 6-month FU visit. (c and d) Close-up of the suspicious lesion on the surface of the right vocal cord.

**Figure 5:**
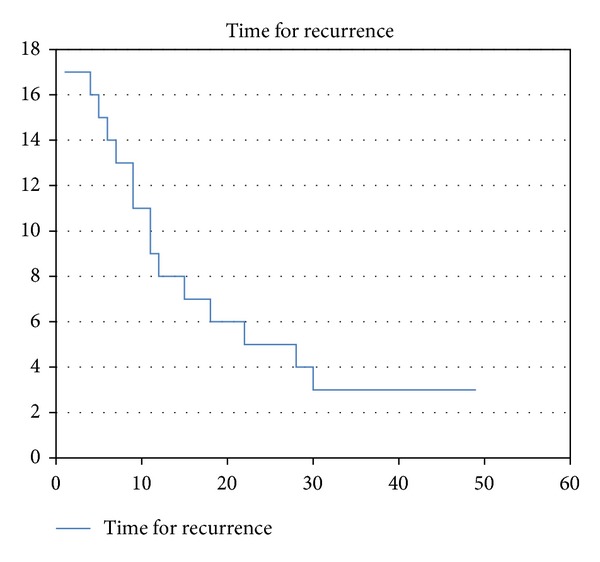
Time to diagnosis of recurrence (*X*—months, *Y*—number of patients).

NBI is an optical method based on the innovative concept of mapping of microvascular changes in superficial layers of mucosa (Figure 1) [14]. The development of these changes in capillary supply is fairly well understood [3]. The method was originally developed for the needs of gastroenterologists as an alternative to intravital staining techniques with different solutions, that is, chromoendoscopy. This methodology has proven beneficial in the area of the esophagus and is also highly applicable in area of hypopharynx, larynx, and lower respiratory system. While the most commonly used staining in the digestive tract is Lugol's iodine solution, in the area of the respiratory tract there is too high risk of aspiration. It is interesting that in the head and neck region it was used for the first time for a screening examination of hypopharynx and larynx in patients previously treated for esophageal cancer [15]. In the last 10 years, there has been a marked increase in the number of publications focused on the evaluation of the accuracy of the examination for the detection of preneoplastic and neoplastic changes in different anatomical regions [16–19]. The most widely used classification describing the character of changes of the capillary loops pattern in tumors was described by Ni et al. [4]. It should be noted that this classification was primarily created for the changes of nonirradiated mucosal surface. We repeatedly encountered the situation that we had observed vascular changes in the mucosa, but that would hardly fit any subgroup of the classification. Therefore, as already mentioned, we must focus on other factors characterizing suspected tumors (ulceration, necrosis, pronounced feeding vessels, an increase in the number of afferent vessels, etc.). Ulceration cannot be assessed as a change better identifiable with NBI mode; it was an additional criterion. We suggest that there is a need for a new classification suitable for irradiated patients that would include the auxiliary criteria and we are currently working on this project. In two works, the topic was directly aimed at detecting cancer recurrences or secondary tumors in patients treated previously with RT or CRT for the head and neck cancer. Piazza et al. selected a group of 59 patients from the database who had previous treatment with RT or CRT [20]. The patients were first examined by WL videoendoscopy that revealed no pathological changes in the IPCL morphology. When using flexible videoendoscopic examination with NBI mode they found suspicious lesions in 13 patients. They always detected typical changes of the microvascular architecture that define a positive finding in the NBI mode. As in our study protocol, all patients subsequently underwent direct endoscopy with the assessment in general anesthesia applying both WL and NBI HDTV magnifying endoscopy. All the suspicious mucosal lesions were successfully removed with free margins and histological findings were correlated with clinical results of preoperative and intraoperative endoscopy. All the methods were confirmed to have very high sensitivity, specificity, and positive and negative predictive value, the highest percentages being found for the combination of NBI and WL HDTV magnifying endoscopy. Their declared values for the flexible videolaryngoscopy with NBI for sensitivity, specificity, and positive and negative predictive value are 100, 98, 92, and 100%, respectively. Patients in this study were treated for tumors of head and neck subsites (the larynx, oropharynx, oral cavity, hypopharynx, and nasopharynx). Quite a large number of them were treated primarily surgically, and RT or CRT was the adjuvant treatment method. Endoscopic examination was considered also a screening method for the identification of the disease in areas other than the localization of the primary tumor. One of the weak points of these studies (we noted similar findings in our study) is the demonstration of true negative findings in the videoendoscopic examination. Repeatedly (consistently) negative findings on repetitive endoscopies are considered true negatives. It is, of course, uncertain whether all of them are actually negative. However, this theoretical premise cannot be demonstrated. More information would provide a longer follow-up of patients, which was shorter than would be needed in all published studies. In the abovementioned study the patients were followed up for a period of 5 to 24 months, with a mean of 10 months. It was an issue also in our study where patient's follow-up was moderately longer (6–48 months, mean 27 months). A similar study was carried out by Japanese authors and published by Lin et al. [21]. In their study 206 patients previously treated for a malignant tumor in the head and neck region were enrolled. 141 of them underwent RT as part of the treatment protocol. The authors have identified 68 mucosal lesions based on flexible transnasal videoendoscopy with NBI mode; 62 cases were histologically confirmed to be a tumor arising from the squamous cell epithelium (Ca in situ or invasive carcinoma). Overall, very high accuracy, sensitivity, specificity, and positive and negative value of the examination were once again demonstrated. Also in their study, patients were monitored for a relatively short period of time; the follow-up was 12–18 months, with a mean of 15.8 months. Piazza's further study summarizes results from a fairly large cohort of patients stratified according to the tumor localization. A very high degree of accuracy of the NBI in detecting malignancies regardless of the localization of the suspicious lesion was proved [22]; the highest diagnostic gain is documented in the area of the oral cavity and oropharynx, where it reaches 25%. In the area of hypopharynx and the larynx the highest accuracy was achieved when intraoperative WL and NBI magnifying endoscopy with angled telescopes were employed. In our study, we confirmed this relationship as flexible endoscopy findings in two of our patients were classified as suspicious, but the examination in WL and NBI HDTV revealed another previously undetected lesion on the mucosa of the larynx. Biopsy of the lesion demonstrated the presence of cancerous pathology. This proves the “added value” of the magnifying endoscopy combined with HDTV. On one hand, the larynx and hypopharynx can be addressed with much greater freedom of movement with the videoendoscope; on the other hand, we often feel safer if we can evaluate the patient with greater ease under general anesthesia. RT or CRT can lead to substantial stiffness in soft tissues, which is unfavorable in terms of direct laryngoscopy. When good exposure of the observed area is obtained, magnifying endoscopy with HDTV becomes an undisputable advantage. The mucosal surface can be observed in close-up view and also under higher magnification. This provides “more pixels on pathology” and gives more information for the evaluation of the lesion. Nevertheless, it is necessary to consider endoscopic approaches as supplementary methods and probably it would be better to determine the effectiveness and accuracy of the NBI method by merging the results of both approaches [18]. The negative histological results of the four patients marked as suspicious in videoendoscopic examination should be comprehensively understood. A considerable number of the patients had surgical attempts to treat the disease endoscopically before definitive RT or CRT. The disease in these patients may have a tendency to submucosal growth and thus may partially conceal the image of microvascular changes. Secondly, it should be kept in mind that even the subjective difficulties of the patient (hoarseness, pain, and discomfort when swallowing) may lead to a clinical suspicion, which might affect the indication criteria for a diagnostic procedure under general anesthesia. The area of the larynx and hypopharynx is less accessible to inspection than, for example, the mucosa of the oral cavity or oropharynx, so physicians tend to be more cautious about possible tumor recurrence. These patients were sent for evaluation under general anesthesia because of the criteria of the study; however, microvascular changes in some of the findings are on the borderline between the precancerous/dysplastic lesion (hyperplasia, mild to moderate dysplasia) and early cancer. In our case, we cannot explain these FP findings by the early phase of learning curve of this method, since the cases were seen throughout the whole course of the study, not only at its beginning. Nonaka published a description of the different character of the microvascular changes (IPCL) in chronic inflammatory processes [23]. In accordance with his conclusions, we observed in irradiated patients general changes in IPCL, but these were diffuse not clearly demarcated and had lower density. The frequency of the detection of cancer in our study remained high, despite the fact that we primarily focused on the area targeted by curative RT and we did not include mucosal lesions from areas other than larynx and hypopharynx. Outpatient office based transnasal videoendoscopic testing was proven as a valid examination of the larynx and hypopharynx even in the presence of late RT-related side effects of treatment.

## 5. Conclusion

The results of the study demonstrate that transnasal endoscopy with NBI in outpatient settings is an excellent method for the follow-up of patients with carcinomas of the larynx and the hypopharynx primarily treated with RT or CRT. It is probably the most significant improvement in the optical diagnostics of recurrent or secondary disease in head and neck cancer patients over the past several decades. A wider availability of the method is still questionable, especially with regard to the relatively high frequency of follow-up visits and costs of the equipment.

## Figures and Tables

**Table 1:** Frequency of follow-up visits.

Frequency of follow-up visits	Year of follow-up			
	1	2	3	4
Frequency of videoendoscopic evaluation	Every 3 months	Every 3 months	Every 4 months	Every 6 months
Frequency of follow-up visits (without NBI)	Every 6–8 weeks	Every 8–12 weeks	Every 4 months	Every 6 months

**Table 2:** Clinical characteristics of the study cohort.

Demography	Patients number (%)
Age, mean, range	62.6 yrs, 42–83
Sex	
Male	44 (66.7)
Female	12 (33.3)
Primary tumor site	
Glottis	56 (85)
T1	35 (62.5)
T2	14 (25)
T3	7 (12.5)
T4	0 (0)
Supraglottis	5 (8.9)
T1	1 (20)
T2	2 (40)
T3	2 (40)
T4	0 (0)
Subglottis	1 (1.8)
T1	1 (100)
Hypopharynx	4 (7.1)
T1	1 (25)
T2	3 (75)
T3	0 (0)
T4	0 (0)
Prior surgery	
Yes	13 (19.7)
No	53 (80.3)

**Table 3:** True positive cases, characteristics.

Number	Sex, age	Pretreatment T, site	Previous treatment	Histology	pT stage	Therapy
1	F, 64	2, G	RT	SCC	3	Endoscopy, waiting for surgery
2	M, 67	2, G	RT	SCC	3	LET
3	M, 64	1a, G	Endo., RT	SCC	3	LET
4	F, 62	3, G	Endo., RT	SCC	3	LET
5	M, 63	2, G	Endo., RT	SCC	3	LET
6	M, 56	1a, G	Endo., RT	SCC	3	FLLE
7	M, 56	1a, G	RT	Ca in situ	1b	Laryngofissure
8	M, 63	1b, G	RT	SCC	3	LET
9	M, 52	1a, G	RT	SCC	2	Endo.
10	M, 52	1a, G	RT	SCC	1a	Endo.
11	F, 60	1a, G	RT	SCC	3	LET
12	M, 71	1a, HP	RT	SCC	1a	Endo.
13	M, 57	3, Sup.	RT	SCC	4	Waiting for surgery
14	M, 62	1b, G	RT	SCC	3	Died before planned surgery

G: glottic, HP: hypopharynx, Sup.: supraglottic, SCC: squamous cell cancer, LET: total laryngectomy, and FLLE: frontolateral laryngectomy.

**Table 4:** False positive cases, characteristics.

Number	Sex, age	Pretreatment T, site	Previous treatment	FU to diagnosis	Histology
1	M, 66	1a, G	RT		Moderate dysplasia
2	M, 42	1b, G	RT		Hyperkeratosis
3	F, 42	1, S	RT		Chronic inflammation
4	M, 57	1a, G	RT		Moderate dysplasia

**Table 5:** Sensitivity, specificity, positive (PPV) and negative (NPV) predictive value, and accuracy of the outpatient NBI videoendoscopy and WL and NBI magnifying endoscopy.

	Sensitivity (%)	Specificity (%)	Positive predictive value (%)	Negative predictive value (%)	Accuracy (%)
Outpatient videoendoscopy with NBI	88	92	76	96	91
WL + NBI magnifying endoscopy	100	92	79	100	94
